# Vitamin D and Autism Spectrum Disorder: A Literature Review

**DOI:** 10.3390/nu8040236

**Published:** 2016-04-21

**Authors:** Hajar Mazahery, Carlos A. Camargo, Cathryn Conlon, Kathryn L. Beck, Marlena C. Kruger, Pamela R. von Hurst

**Affiliations:** 1 Institute of Food Science and Technology—School of Food and Nutrition, Massey University, Palmerston North 4474, New Zealand; h.mazahery@hotmail.com (H.M.); c.conlon@massey.ac.nz (C.C.); k.l.beck@massey.ac.nz (K.L.B.); m.c.kruger@massey.ac.nz (M.C.K.); 2 Department of Emergency Medicine, Massachusetts General Hospital, Harvard Medical School, Boston, MA 02114, USA; ccamargo@partners.org

**Keywords:** review, Autism Spectrum Disorder, ASD, Vitamin D, 25(OH)D, intervention, latitude, ethnicity, migration, season, birth, conception

## Abstract

Low vitamin D status in early development has been hypothesised as an environmental risk factor for Autism Spectrum Disorder (ASD), given the concurrent increase in the prevalence of these two conditions, and the association of vitamin D with many ASD-associated medical conditions. Identification of vitamin D-ASD factors may provide indications for primary and secondary prevention interventions. We systematically reviewed the literature for studies on vitamin D-ASD relationship, including potential mechanistic pathways. We identified seven specific areas, including: latitude, season of conception/birth, maternal migration/ethnicity, vitamin D status of mothers and ASD patients, and vitamin D intervention to prevent and treat ASD. Due to differences in the methodological procedures and inconsistent results, drawing conclusions from the first three areas is difficult. Using a more direct measure of vitamin D status—that is, serum 25(OH)D level during pregnancy or childhood—we found growing evidence for a relationship between vitamin D and ASD. These findings are supported by convincing evidence from experimental studies investigating the mechanistic pathways. However, with few primary and secondary prevention intervention trials, this relationship cannot be determined, unless randomised placebo-controlled trials of vitamin D as a preventive or disease-modifying measure in ASD patients are available.

## 1. Introduction

### 1.1. Description of Autism Spectrum Disorder (ASD)

ASD is a neurodevelopment disorder characterized by impairment in socio-communicative functioning and by limited interests and repetitive and stereotypic behaviours [1]. Depending on the child’s predominant symptomatology, ASD is comprised of different subgroups, including autistic disorder, Asperger Syndrome and Pervasive Developmental Disorder Not Otherwise Specified (PDD-NOS) [2]. ASD usually appears during the first three years of life, but some cases appear later in life when social demands increase (regressive subtype) [1]. Individuals with ASD have: (1) difficulty in expressing and understanding certain emotions; (2) difficulty in understanding others’ mood; (3) impairment in expressive language; (4) abnormal eye contact; (5) prefer minimal changes to routine; and (6) restricted ways of using toys and preference for isolated play, all of which make it difficult for individuals to establish relationships with others, to act in an appropriate way and to live independently [2]. Children with ASD also frequently experience behavioural and medical symptoms. Evidence of abnormalities in inflammatory markers, autoantibodies to brain and glutathione in subsets of ASD patients has been reported; together, these findings suggest that ASD is a systemic condition with the likelihood of being a disease of inflammation, autoimmunity, and/or oxidative stress [3,4,5,6,7,8,9,10,11,12,13,14,15,16].

Over the past few years, the prevalence of ASD has increased dramatically. While previous prevalence studies identified less than 10 in 10,000 individuals [17], recent estimates suggest rates of 90 to 250 in 10,000 individuals [1,18,19,20]. Although this increase is, in part, attributed to the increased awareness and reporting of the disorder, as well as improved diagnostic criteria [21], a complex of genetic and environmental risk factors has also been implicated. It is now believed that ASD is genetically driven and can be triggered by environmental risk factors [22]. In 2008, Cannell published the hypothesis that low vitamin D status, either during foetal life or early childhood, is an important environmental risk factor for ASD [23]. Since then, the role of vitamin D in the aetiology of ASD has drawn epidemiologists’ attention, but intervention research on the role of vitamin D as a preventive and disease-modifying measure is still in its infancy. 

### 1.2. Vitamin D: Metabolism, Biomarker, and Optimum Level

Vitamin D, a fat-soluble vitamin, is a general name for a collection of steroid-like substances including ergocalciferol (D_2_) and cholecalciferol (D_3_) [24]. Vitamin D is present in the diet in limited amounts and is obtained mainly from exposure of skin to UVB radiation. During sun exposure, 7-dihydroxycholesterol which is present mainly in the layers of epidermis and to a lesser extent in dermis [25] absorbs UVB radiation and is converted to pre-vitamin D_3_ [24]. Then pre-vitamin D_3_ undergoes heat-induced isomerisation and forms vitamin D_3_. Vitamin D_3_ is derived from a cholesterol metabolite, and vitamin D_2_ is the product of irradiation of plants [24]. Vitamin D binding protein (DBP) in the circulation is responsible for the transport of both vitamins D_2_ and D_3_. Carried by DBP, these vitamin D metabolites enter the liver where they are hydroxylated and converted to their respective 25-hydroxyvitamin D metabolites (e.g., 25(OH)D_3_) [24]. These metabolites are inactive until they are converted to 1,25-dihydroxyvitamin D [1,25(OH)_2_D] by 25-hydroxyvitamin D-1α-hydroxylase (1-OHase), and become biologically active [26]. At this point, vitamin D can bind to the vitamin D receptor (VDR)—found in most body tissues and organs [24,26]—and exert its musculoskeletal and non-musculoskeletal role in the body. However, because 1,25(OH)_2_D synthesis is tightly regulated, circulating 25(OH)D has been suggested to be the best single indicator of vitamin D status; it reflects both dietary vitamin D intake and vitamin D synthesised by UVB radiation [27,28].

Vitamin D deficiency and insufficiency are controversial terms but generally defined as serum 25(OH)D concentration <25 nmol/L and 25–49.9 nmol/L, respectively [29]. Other cut-offs for vitamin D deficiency and insufficiency are used by other groups, e.g., <50 and 50–74.9 nmol/L, respectively [30]. Until there is more clarity on these terms, we believe it is best to report the actual values rather to rely on ambiguous terms.

### 1.3. Objective

We aimed to investigate the relationship between vitamin D and ASD, and to systematically review the literature for all studies on this relationship, including studies on the mechanistic pathways that might underlie this relationship. 

## 2. Methods

Firstly, we reviewed previous reviews [23,31,32] to identify vitamin D-related risk factors related to ASD. We grouped the identified factors into seven specific areas including (1) latitude; (2) season of birth and conception; (3) maternal migration and ethnicity; (4) vitamin D status of mothers; (5) vitamin D status of ASD patients; (6) maternal vitamin D intervention to prevent ASD; and (7) vitamin D intervention to treat ASD. According to their relationship with prevention stages, each of these areas were assigned to one of the three major areas of; (1) vitamin D-ASD-related areas providing indications for primary prevention; (2) vitamin D-ASD-related areas providing indications for both primary and secondary prevention; and (3) vitamin D-ASD-related areas providing indications for secondary prevention. 

We performed a literature search covering studies published up to January 31 2016 in PubMed, the Web of Knowledge, EBSCO OVID, MEDLINE, PsycARTICLES, PsycINFO, SocINDEX and Google Scholars databases. Because it was impossible to cover all specific areas with one fixed term search we employed a distinct search strategy for each specific area. The search strategy was as follows: autism or Asperger or “Autism Spectrum Disorder” or ASD or “Pervasive Developmental Disorder” or PDD AND the following distinct search terms for each specific area;

- “incidence” or “prevalence” to search the literature in relation to latitude. For studies published between 1992 and 2012, we identified literature from a previous systematic review of autistic disorder and PDD prevalence worldwide [33] to have a manageable data for search. We excluded studies published before 1992 because case ascertainment was based on DSM-III and ICD-9.
- “season” or “month” AND “birth” or “conception” to search the literature in relation to Season of conception or birth.
- “migrant” or “immigrant” or “migration” or “immigration” AND “maternal” or “mother” to search the literature in relation to maternal migration and ethnicity.
- “vitamin D” or ergocalciferol or “vitamin D2” or “cholecalciferol” or “vitamin D3”or “25-hydroxyvitamin D” or “25(OH)D” or “25ohd” to search the literature in relation to maternal vitamin D status, vitamin D status in ASD patients, maternal vitamin D intervention to prevent ASD, and vitamin D intervention to treat ASD.

We followed this summary with a presentation of the mechanistic pathways by which vitamin D may exert its role in either aetiology or pathobiology of ASD. The selection criteria for each of these specific areas varied and, for maximal clarity, the criteria are presented at the beginning of each specific area. 

## 3. Three Major Areas of Research

### 3.1. Vitamin D—ASD-Related Areas Providing an Indication for Primary Prevention

In this section, we will discuss vitamin D-related risk factors related to ASD which can be targeted for preventive purposes before ASD occurs. We found five specific areas including latitude, season of conception and birth, maternal migration and ethnicity, vitamin D status of mothers, and maternal vitamin D intervention to prevent ASD.

#### 3.1.1. Risk of ASD—Latitude

We included studies that met the following criteria: (1) reported a prevalence estimate for either of the following: Autistic Disorders (AD), ASD (including AD, Asperger’s Syndrome, PDD-NOS and PDD (ASD plus Rett’s disorder and Childhood Disintegrative Disorder); (2) case ascertainment was based on either Diagnostic and Statistical Manual of Mental Disorders—Fourth Edition (DSM-IV) or International Classification of Diseases and related health problems—Tenth Edition (ICD-10); and (3) included children <8 years of age. Studies that used medical registry or records only were excluded. There are several methodological factors that affect the prevalence estimates of ASD and these include classification systems, case ascertainment and participants’ age. In studies using DSM—III, the prevalence is much lower than in those using DSM-IV or the Chinese classification system of Medical Disorders (CCMD) [34]. Also, some individuals previously diagnosed with ASD according to DSM-IV might miss out on a diagnosis with the new criteria outlined in DSM-5 because the definition of autism has gone through three major changes [35]. Several separate categories in DSM-IV including AD, Childhood Disintegrative Disorder, Asperger’s Syndrome and PDD-NOS has been included into the one overarching category named “ASD”, the three domains of impairments in social interaction, impairments in communication and stereotyped behaviour in DSM-IV have become two, with the merging of the first two domains (deficits in social-communicative functioning), and finally the diagnosis age of three has been dropped and included manifestation of disorders either currently or by history [2].

Our literature search identified prevalence data for eight countries [17,36,37,38,39,40,41,42,43,44,45]. Four studies were from Asia (latitudes 24.46–39.91°N) [36,42,43,45], one from South America (34.28°S) [44] and six from Europe (51.24–63.91°N) [17,37,38,39,40,41]. We did not identify any study from the US, Africa and Australia/New Zealand that met our inclusion criteria. Among the included studies, different tools were employed, including eight screening instruments and seven diagnostic tools. There were seven studies that reported prevalence estimate for AD [17,36,37,38,39,40,41], four for PDD [42,43,44,45] and three for both AD and PDD [17,37,38]. Data regarding the prevalence estimate for AD and PDD are presented in Table 1, respectively. The AD prevalence estimate per 10,000 ranged from 10.9 in China [36] to 60.0 in Sweden [40]. The PDD prevalence estimate ranged from 6.3 in Iran [43] to 181.0 in Japan [45], and, on average, was >30 in Europe. 

Two systematic reviews of epidemiological studies illustrated a regional variation in the prevalence estimate for autism [46], and a four-fold increase in the prevalence estimate for infantile autism in moving from Israel to Sweden latitudinally [47]. Consistent with these findings, we also observed a latitudinal variation in the prevalence estimate for AD and PDD, with those in the lower latitudes reporting lower prevalence estimates and vice versa. There were, however, a few exceptions; compared to Sweden [39], Finland had lower AD prevalence estimate despite being located at the quite similar latitude [41]. Mandatory vitamin D food fortification of certain foods and dietary supplementation policies may have improved vitamin D status in Finish populations [48,49,50], and may explain the lower prevalence estimate for AD in this country. The higher prevalence estimate for PDD in Japan compared with European countries may also, in part, be explained by the higher prevalence of low vitamin D status among Japanese women than European women. Approximately 95% of Japanese pregnant women have been reported to have 25(OH)D levels <50 nmol/L; the corresponding figures in the UK and Latin America have been reported to be 55% and 37%, respectively [51,52], a finding confirmed by a systematic review of global maternal and new born vitamin D status [53]. This may be due to lower dietary vitamin D intake and more sun—avoidance behaviour in Japanese population [54].

The latitude differences in prevalence estimates for autism should be interpreted with caution since they assume that latitude is a reasonable proxy measure for vitamin D status. If low vitamin D status is a risk factor for autism, it is expected to see high prevalence of autism in countries with high prevalence of low vitamin D status and vice versa. This relationship is well illustrated by both low prevalence estimates for PDD and vitamin D deficiency/insufficiency in Argentina (34.28°S). The prevalence estimate for PDD was 13.1 per 10,000, and for 25(OH)D levels <25 and <50 nmol/L was reported to be only 3% and 33% in young children and adults, respectively [55]. 

However, the low prevalence estimate for AD in China and PDD in Iran and UAE despite having high prevalence of low vitamin D status [56,57,58] may be due to methodological issues (screening and diagnostic characteristics) and underreporting. Translation of a screening or diagnostic instrument may adversely affect its psychometric properties and therefore its sensitivity in picking up the cues for autism [43]. This is an issue for non—English speaking developing countries such as Iran and UAE. Employing different screening cut-off ranges can also be a driving factor; using a wider cut-off range includes more children for the final diagnosis and thus fewer children are likely to miss the diagnosis. Furthermore, a shortage of service delivery, stigmatising effect of autism on parents, parental tolerance and expectations of children’s behaviour and cultural influences and norms for atypical behaviours may be associated with underreporting of autism in developing countries [59].

Within each study population, it is also important to consider UVB doses reaching the population, differences in skin pigmentation, sun—avoidance or seeking behaviours, clothing style, and racial and ethnic differences. The prevalence of infantile autism in cohorts born before 1985 was reported to be in the earlier months to the North, perhaps partly, it is associated with the lower UVB doses and therefore lower vitamin D production [47]. Grant *et al.* (2013) reported an inverse relationship between the solar UVB radiation doses and the prevalence of ASD [60]. Accordingly, living at higher latitudes might be associated with lower doses of UVB radiation, if not compensated by more sun exposure or ingestion of vitamin D rich foods or supplements, and consequently increased risk of ASD.

#### 3.1.2. Risk of ASD—Migration and Ethnicity

We included studies that (1) reported odds ratio (OR), prevalence ratio or relative risk for AD, ASD and/or PDD; (2) reported mother’s country of birth or race/ethnicity; and (3) case ascertainment was based on formal diagnoses according to disease classification systems. 

Our literature search identified 10 studies [61,62,63,64,65,66,67,68,69,70], with seven studies from Europe [61,62,63,64,65,67,70], two from the US [68,69] and one from Australia [66] (Table 2). The number of cases ranged from 250 in a study from Sweden [67] to 7540 in a study from the US [69]. Depending on the study design, statistical analysis were adjusted for a range of confounding factors, ranging from only one factor [63] to several factors in other studies [61,62,64,65,66,67,68,69,70], though the same factors were not considered by all studies.

Most studies showed increased risk of ASD among children of migrant parents [61,62,63,64,65,66,67,69,70], while some reported no association [61,65,68,69] or even decreased risk [61,68,69,70]. Williams *et al.* (2008) reported that the odds of having a child with ASD among mothers born outside Australia significantly increased compared with those born in Australia, 1.4 (95% CI, 1.0–1.9; *p* = 0.009) [66]. The mothers’ original countries were not reported. Keen *et al*. (2010) reported an adjusted OR ranging from 1.2 (95% CI, 0.8–1.9) in children of European mothers born outside the UK to 10.0 (95% CI, 5.5–18.1) in children of Caribbean mothers compared with mothers born in the UK [63]. Magnusson *et al.* (2012) also reported an increased risk of having a child with ASD among mothers born outside Sweden as compared to those born in Sweden [61].

Magnusson *et al.* (2012) reported an increased risk of low functioning autism and decreased risk of high functioning autism in children of migrant parents in Sweden, 1.2 (1.0–1.4) and 0.5 (0.4–0.6), respectively [61], a finding confirmed by Williams *et al.* 2008 [66] and Becerra *et al.* (2014) [69]. The risk of having a child diagnosed with both autism and mental retardation increased two-fold in foreign-born black, Vietnamese and Filipino mothers [69]. In a study from the US, the proportion of children with ASD with intellectual disability was significantly different between black, Hispanic and white children: 48%, 38% and 25%, respectively [71]. A possible explanation is that mothers with darker skin are at increased risk of low vitamin D status and moving to northern latitudes with lower solar UVB radiation may exacerbate the condition and thereby lead to more severe disability among their children. It should be pointed out that having 25(OH)D levels in this study would address that explanation. 

van der Ven *et al.* (2013), however, reported a decreased risk for ASD in children whose mothers were born in developing countries compared with those born in developed countries [70]. Within the ASD subtypes, a significant increased risk for AD and a decreased risk for Asperger Syndrome and PDD-NOS combined was reported in mothers of developing countries. These findings are consistent with several other studies reporting an increased risk for AD [62,64,65,67,68,69] and decreased risk for Asperger Syndrome [67] in children of mothers born outside the reference country. These findings are mirrored in studies investigating the effect of child’s ethnicity on prevalence estimate of ASD. While children of black ethnicity had increased risk for AD, 2.6 (1.3–5.0), the risk for Asperger Syndrome and PDD-NOS decreased in these children 0.5 (0.2–1.0) [72]. An explanation is that the aetiology of AD might be different from those of Asperger Syndrome and PDD-NOS in different race/ethnic group and that low vitamin D status which is highly prevalent in some race/ethnic groups, could exacerbate the condition, and cause AD.

The question remains why migration status is associated with no effect or decreased risk in some studies. Those showing no or decreased effect of maternal migration status mainly compared the prevalence estimates for autism among mothers born in the same region or of white ethnicity [61,64,65,69]. These populations share quite homogeneous characteristics with those of the reference country—the majority, if not all, have white skin colour and quite similar culturally accepted dressing code and attitudes toward sun exposure—that may affect vitamin D status of these populations.

The risk of AD was reported to be higher in children of mothers born in Mexico and in East Asian countries living in the US than those of mothers born in the US [68,69]. The decreased association reported in these studies may, in part, be attributed to the statistical analysis and the characteristics of minority groups. Although the relative risk of having a child with AD in mothers born in Mexico was 0.6 (95% CI, 0.5–0.7) in the Croen *et al.* study [68], it was 1.1 (95% CI, 1.0–1.2) in another study by Becerra and colleagues [69]. In the latter study, statistical analysis was adjusted for more variables (13 *vs.* 7 variables, respectively), including diagnostic variability [68,69]. It is well documented that diagnostic variability contributes significantly to the prevalence estimates [34]. The effect of covariates on final findings is also illustrated by the lower crude rates for ASD in black and US-born Hispanics that was similar or slightly higher than the US-born whites when the statistical analysis was controlled for several covariates [69]. Furthermore, minorities might be under-identified for ASD due to lower socioeconomic status, cultural differences in views of typical and atypical behaviours, viewing communication and social skill delays as temporary that disappear with age, parent-health professional communication gap and lack of culturally sensitive screening and assessment tools [73]. Finally, not all studies controlled for sun exposure variables; therefore it is difficult to relate these results directly to vitamin D-ASD relationship.

#### 3.1.3. Risk of ASD-Season of Conception and Birth

We included all studies that relied on formal diagnoses of autism (according to disease classification systems) and reported season/month of conception or birth in patients with autism (infantile autism, AD, and ASD). 

Our literature search identified 20 studies (Table 3); with one study reporting the season/month of conception only [74], three reporting both the season/month of conception and birth [75,76,77], and 16 reporting the season/month of birth only [64,78,79,80,81,82,83,84,85,86,87,88,89,90,91,92]. The number of cases ranged from 54 in a Turkish study [79] to 19,328 in a US study [75]. Most participants in the control groups were live births (13 studies), two included siblings [78,85], one, both siblings and live births [89], three healthy age and gender matched children [78,79,81], and one compared cases with other neuro-developmental disorders [86]. Most of these studies did not control for any covariates in the statistical analysis [76,78,79,81,84,86,87,88,89,91,92]. Eight studies made adjustments for covariates ranging from two [82,83] to 12 covariates [64], though different factors were considered in these studies. 

Children conceived during the winter months, when sun exposure is limited and vitamin D status is lower, were reported to have a higher risk of developing autism later in life than those conceived during the summer months [74]. Masumdar *et al.* (2012) [74] reported an increased rate of children later diagnosed with AD conceived in winter (final three weeks of November and first weeks of December) in California from 1994 to 1996 (*p* < 0.05), though the seasonality finding disappeared between 1996 to 2000. The authors did not control for maternal residence at birth. While controlling for several variables including year of conception and maternal residence at birth, Zerbo *et al.* (2011) reported a 6% (OR 1.06, 95% CI 1.02–1.10) increase in the risk of AD in children conceived during winter compared with summer months in California between 1990 and 2002 [75]. The incidence rate of AD increased steadily from the conception month of August to March, decreased from March to April and then remained unchanged from April to July. The rate of AD in November births was significantly higher compared to April. 

By contrast, Hebert *et al.* (2010) reported an increased rate of children later diagnosed with ASD in those conceived during summer in the UK; with a conception rate of 9.5/1000 *vs.* 5.7/1000 in summer (June—August) and winter (December-February) months, respectively, with a corresponding peak in spring births, an OR of 1.86 (1.01–3.37) [76]. The study had a small sample size of 86 cases, and the age, duration and follow up measures were not clearly stated. Moreover, there was no adjustment for potential confounding. 

Lastly, Atladottir *et al.* (2007) found no association between season of conception or birth on incidence rate of ASD and ASD subcategories in a cohort born in Denmark between 1990 and 1999 [77]. The sample size was smaller (*n* = 1860) than the sample size in the Masumdar *et al.* and Zerbo *et al.* studies (*n* = 8074 and 19,328, respectively), and the authors did not control for several variables included in the latter studies. 

Season/month of birth and risk of autism has also been a research focus among epidemiologists. Spring birth has been associated with increased risk of autism in studies conducted in Canada [92], Japan [91], the US [82], Sweden [78,90], and the UK [76]. March birth as a risk factor for autism has also been reported by several studies from Canada [92], Denmark [80,88], the US [85] and Israel [87]. Lee *et al.* (2008) reported three peaks during spring, summer and autumn months in singleton and multiple births concordant with ASD (*n* = 1051), and an 87% decreased risk for multiple births concordant for ASD in males in winter (December) compared to other seasons [relative risk 0.18, 95% CI 0.04, 0.82; *p* < 0.05) [82]. Consistent with these findings, Konstantareas *et al.* (1986) and Gillberg *et al.* (1990) also reported a significant deviation of season/month of autistic births from that of the general population among males, a pattern that was not evident in females (*n* = 36 and 25, respectively) [90,92]. Konstantreas *et al.* commented that this finding could be due to the sampling artefact. With a male: female ratio of 4:1 and an ASD multiple birth sample size of 161 [82], the results related to gender differences in the Lee *et al.* study (2008) may also be a sampling artefact. 

In contrast, Maimburg *et al.* (2010) reported increased risk for infantile autism in those born in winter (October to March) [80]. Aggregation of different months into seasons or half years can result in different results or even loss of information. Bolton *et al.* (1992) reported different results by moving one month from a season and including it in the next month, for instance inclusion of December in the winter or autumn months [89]. A seasonal effect of birth on autism was reported when December was included in the winter; however, this pattern disappeared when December was included as an autumn month. As such, inclusion of both December and March in winter in the Maimburg *et al.* study (2010) [80], may explain the increased rate of autism births during this season. 

Not all studies showed an association between season of birth and risk of autism [64,77,79,81,83,84,85,86]. The absent association in these studies may be attributed, at least in part, to the definition of season (discussed previously). Hultman *et al.* (2002) [64] categorised months into two seasons; four months in one season *versus* eight months in other season, a method that may have obscured the association. Furthermore, the small sample size in the Meguid *et al.* (2010), Ugar *et al.* (2014) and Fernell *et al.* (2015) studies (all *n* < 70) may have contributed to their non-significant findings. 

The characteristics of the populations included in case and control groups, and case ascertainment may also mask the seasonal variability among groups. For instance, Fennell *et al.* (2015) did not find any seasonal deviations for ASD populations from the typically developing controls of Middle Eastern/African origin, though the season of birth difference was significant in cases and controls of Swedish origin (38% in spring *vs.* 18% in summer; *p* < 0.05) [78]. This could be due to the narrow range and low year-round mean 25(OH)D concentrations in these populations (28.5–33.3 nmol/L in winter and summer, respectively) [93]. Kolevzon *et al.* (2006), identified cases through a screening programme for military services in Israel and Orthodox women were exempted from the military service. Therefore, some autistic cases might have been missed [83]. Landau *et al.* (1999) included cases with autism from different regions with different environmental influences and controls were individuals with mental retardation [86]. Disorders of neurodevelopment may share the same environmental influences and inclusion of these disorders in the control group may mask the seasonal variability among groups. 

Interestingly, some studies did not find a season/month of birth effect on aggregated samples but reported seasonal deviations from the general population in autism with comorbidities such as language and developmental delay [84,85]. For example, Yeates Frederiks *et al.* (2000) did not find an effect of season of birth on autism rate in the aggregated sample, but they found a significant seasonal trend for low functioning autism (autism with IQ < 35) (increased rates for autistic births in the second quarter of the year) [84]. This reflects a trend similar to the Konstantreas *et al.* (1986) [92] and Stevens *et al.* (2000) [85] findings of a more pronounced seasonal effect on low functioning and or nonverbal autism. The differences in the seasonal effect of birth and risk of low and high functioning autism might be attributed to different etiological factors or to the same etiological factors but of different intensity. Whitehouse *et al.* (2012) reported a significant positive relationship between the severity of maternal vitamin D deficiency and the severity of the language impairment (mild and moderate to severe) in children at five and 10 years of age [94]. As such, the lack of season of birth variation does not exclude the possibility of an association if autism with comorbidities was considered. 

Finally, seasonal of birth is a crude proxy measure for sun exposure and, more specifically, vitamin D status; many other potentially relevant factors vary by season. These environmental factors include application of pesticides [95], infection [96], nutritional factors [97] and maternal biochemistry [98], all of which have been hypothesised to be implicated in the aetiology of ASD, and need to be taken into consideration when interpreting the literature on season of conception/birth and risk of ASD.

#### 3.1.4. Risk of ASD-Vitamin D Status in Mothers

We included all studies reporting maternal vitamin D status and the risk for AD, Asperger Syndrome and or ASD in offspring. Our literature search identified two articles investigating the relationship between maternal vitamin D status and autism among offspring. Fernell *et al.* (2010) compared maternal 25(OH)D concentration of Swedish mothers with and without a child with autism (*n* = 12 with vs 14 without) and Somali mothers with and without a child with autism (*n* = 14 with vs 17 without) in autumn and spring [99]. More than 60% and 85% of mothers of Somali origin had 25(OH)D <25 nmol/L in spring and autumn, respectively, compared to only one mother of Swedish origin in both seasons. The difference in 25(OH)D concentrations was not significantly different between mothers with and without a child with autism for either ethnicity. This study is limited, however, by its small sample size and the long time period since pregnancy (average age of more than 4 years). Furthermore, a lack of difference could be also attributed to the very low and narrow maternal 25(OH)D concentration among mothers of Somali origin which does not allow the relationship between vitamin D status of mothers and risk of ASD to be addressed.

To overcome these limitations, Whitehouse *et al.* (2013) investigated the relationship between maternal 25(OH)D concentrations at 18 weeks of pregnancy and autism phenotype in the offspring at ages 5, 8, 10, and 14 years follow ups [100]. The authors did not find any difference in 25(OH)D concentrations among mothers of children with and without clinical ASD (*n* = 929) while controlling for several variables (maternal education, socioeconomic status, maternal race, age at conception, maternal smoking, alcohol intake during pregnancy, parity, infant health and sex). It should be noted that only three males were clinically diagnosed with ASD at follow up and extrapolating these results to the broader ASD population should be done with caution. Maternal 25(OH)D concentrations were not associated with the total score and four out of five subscales of Autism Like Quotient (autistic-like traits) among 406 offspring. However, compared to children of mothers in the higher tertiles, children of mothers in the lower tertile (25(OH)D <45 nmol/L) had higher scores on attention switching subscale (OR 5.46, 95% CI, 1.29–23.05, *p* < 0.05) while controlling for several potential confounders, including season of blood collection. A limitation of this study is that only 14% of offspring from the original cohort completed the study. The high attrition rate was biased toward the loss of mothers with lower socioeconomic status. It was shown that total autistic like trait score was higher among offspring of mothers with low socioeconomic status and of mothers with low education levels. On the other hand, evidence shows that both lower socioeconomic status and lower maternal education are associated with lower vitamin D status [101,102].

Consistent with these findings, Whitehouse *et al.* (2012) and Morale *et al.* (2012) also showed that lower maternal 25(OH)D concentration in pregnancy (weeks 18 and 13.5, respectively) was associated with more language difficulties and mental and psychomotor outcomes in offspring, respectively [94,103]. While controlling for several variables, maternal 25(OH)D concentration <46 nmol/L was associated with significant language difficulties in the offspring at ages 5 and 10 years compared with those offspring of mothers with 25(OH)D concentrations >70 nmol/L [94]. Maternal 25(OH)D concentration >75 nmol/L in pregnancy was associated with increased mental and psychomotor scores in infants at 14 months compared to those with 25(OH)D concentrations <50 nmol/L [103]. 

By contrast, Gale *et al.* (2008) could not find any relationship between maternal 25(OH)D concentration during pregnancy and child’s intelligence and psychological health at ages 9 months and 9 years [104]. The inconsistent findings could be, in part, attributed to important methodological issues and the timing of blood collection during pregnancy. At the follow-up, the sample size was smaller (*n* = 178) and the attrition rate was higher (70%) in the Gale *et al.* study [104] than the Whitehouse *et al.* and Morales *et al.* studies (*n* = 412 and 50% and *n* = 1826 and 25%, respectively) [94,103]. Maternal 25(OH)D concentrations during pregnancy and infants’ neuropsychological health might have differed between the many lost to follow-up and this could have biased the results. 

Furthermore, while the blood sampling was done in late pregnancy (week 32.6) in the Gale *et al.* study [104], blood samples were collected during weeks 18 and 13.5 ± 2.1 of pregnancy in the Whitehouse *et al.* and Morales *et al.* studies [94,103], respectively. These findings suggest that depending on the timing of the exposure to low vitamin D status, different brain areas might be affected and the consequence might be different neurodevelopment and cognitive outcomes in infants [105].

#### 3.1.5. Vitamin D Intervention to Prevent ASD

We included all studies reporting using vitamin D supplements during or before pregnancy in relation to ASD in the offspring. We excluded those studies using vitamin D containing multivitamins or minerals only because those doses are relatively low and the effect of vitamin D *per se* cannot be determined from these interventions.

Our literature search identified only one study reporting on the effect of vitamin D supplementation during pregnancy on the recurrence rate of autism in newborn siblings [106] (Table 4). In an open-label prospective study, Stubbs, Henley and Green (2016) prescribed vitamin D at a daily dose of 5000 IU to mothers of children with ASD during pregnancy (majority during the second trimester, *n* = 19), 7000 IU during breastfeeding, and 1000 IU to their newborn infants during the first three year of life if they were not breastfed. The authors investigated the recurrence rate of autism in these children and compared it to other literature. The recurrence rate of autism was approximately four-fold less than that of other studies (5% *vs.* 20%). To note, this study had a small sample size and lacked a control arm. 

### 3.2. Vitamin D-ASD-Related Areas Providing an Indication for both Primary and Secondary Prevention

In this section, we will synthesise the literature regarding a vitamin D-related measure—that is low vitamin D status in ASD patients—which can be a risk factor for ASD, and at the same time, a stage of ASD disease course.

#### Vitamin D Status in ASD Patients

We included all case-control studies of vitamin D status of individuals with ASD compared to healthy individuals, and participants of any age diagnosed with an ASD by either established diagnostic criteria, for example, DSM-IV or standardised established instruments. 

Our literature search identified 16 studies reporting 25(OH)D concentrations in populations with ASD [78,79,81,107,108,109,110,111,112,113,114,115,116,117,118] (Table 5). Two of the 16 studies were excluded because either the full article was in Chinese [107], or cases were not compared with typically developing controls [119]. All 13 studies included in this review have been published over the past five years. Approximately half of the literature was from the Middle East [79,81,109,111,115,118], two from China [112,117], two from Europe [78,108], three from the US [113,114,116] and one from South America [110].

The number of cases ranged from 13 [115] to 254 [118]. Fernell *et al.* (2015) included siblings as controls [78], Saad *et al.* (2015) [111] and Kocovska *et al.* (2014) [108] included siblings, parents and typically developing children as controls, and the remaining studies included only non-family member typically developing children. Children included as controls were thoroughly examined for medical and/or mental and developmental conditions in three studies [81,111,112]. Controls were matched to cases on one or more attributes, including age, sex, ethnicity, socioeconomic status, season of birth, and/or season of blood collection. Season of blood collection was controlled in eight studies by either making adjustments in statistical analysis [108,113,114], performing all blood collections in one or two seasons [109,111,117], matching controls to cases on season of enrolment [79] or performing sub analysis for different seasons [112].

Of 14 studies, nine reported significant differences in 25(OH)D concentrations between cases and controls [78,81,108,109,110,111,112,117,118], one borderline difference [114], and four found no significant differences among study groups [79,113,115,116]. Tostes *et al.* (2012) reported that mean 25(OH)D concentration of children with ASD of different ethnic groups was lower by approximately 35 nmol/L than healthy age and sex matched controls (*p* < 0.001) [110]. Neither ethnicity nor season of blood sampling was considered in the statistical analysis. 

Bener *et al.* (2014) investigated several lifestyle and biochemical risk factors in relation to autism [118]. Children with ASD had significantly lower 25(OH)D concentrations than healthy age, sex and ethnicity matched controls (46.0 ± 20.5 *vs.* 54.0 ± 21.0 nmol/L, respectively, *p* = 0.004). Circulating levels of 25(OH)D were inversely correlated with autism severity measured by Autism Diagnostic Observation Schedule (ADOS). Factors associated with autism were serum calcium, 25(OH)D, blood relative, BMI, physical activity, child order, and ferritin. Despite the large sample size of 254 in each group, this study had several limitations; (1) cases were selected from a cohort study and controls were not examined for autism manifestations; (2) potential covariates or confounders were not controlled for in statistical analysis despite significant differences in socio-demographic characteristics of cases and controls; and (3) season/month of blood sampling was not controlled for in statistical analysis.

Using siblings or family members as controls to minimize confounding by socio-demographic factors, Fernell *et al.* (2015) reported significantly lower mean 25(OH)D concentration at birth in autistic children of Scandinavian and other European origins than their non-ASD sibling controls [78]. On the other hand, the authors did not find any differences between cases and controls of Middle Eastern/African origin. The lack of association in Middle Eastern and African group may in part, be attributed to the small sample size and very low mean 25(OH)D concentrations in these two ethnic groups, 11.5 ± 7.1 and 7.0 ± 5.0 nmol/L, respectively. It should be also noted that season of birth was not significantly different between cases and controls in the latter ethnic groups, but it was significantly different across groups of Scandinavian and other European origins (10% *vs.* 35% of cases and controls in summer, respectively). Season of birth which corresponded to the season of blood sampling in this study was not controlled for in statistical analysis. 

Kocovska *et al.* (2014) showed that ASD individuals had significantly lower 25(OH)D concentrations than their siblings, parents and healthy controls while adjusting for season of blood sampling (no one in ASD group had his/her blood sample drawn in winter) [108]. Furthermore, a larger proportion had 25(OH)D <50 nmol/L in cases than controls (88% in ASD group *vs.* 58% in sibling and parents each and 65% in healthy controls). A limitation to those studies including family members is that cases and controls cannot be matched on age and sex. Furthermore, inclusion of older siblings may confound the findings by younger parental age, and inclusion of apparently healthy younger siblings, on the other hand, may run the risk of including siblings with ASD who may develop ASD later in life in the control group [78,108]. For that reason, it is important that investigators examine family member controls more carefully for any autistic-like behaviour. 

Examining both cases and controls for autistic-like features, Gong *et al.* (2013) reported a larger proportion of children with ASD having 25(OH)D concentrations <50 nmol/L as compared to healthy controls (58% *vs.* 33%, respectively *p* = 0.01) [112]. Autism severity—measured by Childhood Autism Rating Scale (CARS)—negatively correlated with 25(OH)D concentrations (*r* = −0.4, *p* < 0.001) while controlling for several covariates including age, sex, season, magnesium, BMI, calcium and phosphate. When vitamin D status of study groups was compared according to the four seasons, children with ASD had lower summer 25(OH)D concentrations than controls (*p* = 0.02). It should be noted that doing sub-analysis in studies with small sample size results in even smaller sample sizes for each group, which could compromise the validity of the latter findings. As a general rule, it’s preferable to control for season of blood sampling either in the statistical analysis or by limiting season/month of blood collection rather than doing this by subgroup analysis, especially in studies with a small starting sample size.

Meguid *et al.* (2010) [81] and Saad *et al.* (2015) [111] also thoroughly examined all children, including both cases and controls, for medical conditions and mental disorders, and reported that two groups of Egyptian children with ASD had significantly lower levels of 25(OH)D compared to age, sex and socio-economic status matched healthy controls. The season of birth was taken into account in both studies but no significant differences were found in either group. Mean 1,25(OH)_2_D and calcium concentrations was also lower in children with ASD than healthy controls [81], and serum 25(OH)D concentrations negatively correlated with CARS scores (*r* = −0.50, *p* < 0.0001) [111]. Children on the severe end of the spectrum had lower mean 25(OH)D concentration than those with mild/moderate ASD (30 ± 15 *vs.* 53 ± 21 nmol/L, respectively). To avoid seasonal variations in 25(OH)D concentrations, all blood samples in this study were drawn during same two months (May and June).

Similarly, Mostafa and Al-Ayadi (2012) collected all blood samples from Saudi Arabian children with ASD and healthy controls during summer months [109]. Children with ASD displayed significantly lower 25(OH)D concentration than healthy controls. While 40% of children with ASD had 25(OH)D concentrations <25 nmol/L, no one from control group had 25(OH)D concentrations in that range. Cases also had significantly higher anti-myelin-associated glycoprotein (anti-MAG) anti-autoantibodies than controls. Furthermore, 25(OH)D concentrations correlated negatively with CARS scores, (*r* = −0.84, *p* < 0.001) and anti-MAG anti-autoantibodies (*r* = −0.86, *p* < 0.001). Children on the severe end of the spectrum had significantly higher anti-MAG anti-autoantibodies levels than those with mild/moderate ASD. These findings point to a link between low vitamin D status, autoimmunity and autism.

In a recent large study from China, Feng *et al.* (2016) reported higher proportion of ASD children having 25(OH)D concentrations <25 nmol/L (13% *vs.* 0%, *p* = 0.03) and <75 nmol/L (71% *vs.* 62%, *p* = 0.03) than healthy typically developing controls [117]. To control for seasonal variation, the study lasted for six months; April—September corresponding to spring and summer months. To note, 25(OH)D concentrations of those entering the study in April could be different of those entering September due to a potentially limited sun exposure during winter months.

Neumeyer *et al.* (2013) [114] reported higher proportion of boys with ASD having 25(OH)D concentrations <80 nmol/L than healthy boys (77% *vs.* 37%, *p* = 0.02). Furthermore, cases had marginally lower serum 25(OH)D concentration than controls (67 ± 5 *vs.* 79 ± 4 nmol/L, *p* = 0.05). Despite comparable total energy intake and caloric intake from specific macronutrients among study groups, calcium and vitamin D intake was lower in cases than controls. Small sample size was a limitation of this study (*n* = 18) with the sample size calculated based on differences in bone mineral density rather than 25(OH)D concentrations. 

Hashemzadeh *et al.* (2015) failed to show any difference in 25(OH)D concentrations between children with ASD aged 3–12 years (*n* = 13), and age and sex matched typically developing children (*n* = 14) [115]. This study had the smallest sample size, a limited statistical power to detect differences among groups. Three other studies that also failed to show any relationship had somewhat larger sample sizes (ranging from 54 to 71 per group) and controlled for several covariates [113] and matched controls to cases on several attributes [79,116]. Molloy *et al.* (2010) [113] included boys with catheters placed for tonsillectomies as controls. Children were suffering from some form of acute inflammation, which potentially had an effect on 25(OH)D levels [120,121]. 

Although Adams *et al.* (2011) reported statistically significant differences in several biomarkers indicative of increased oxidative stress, reduced capacity for detoxification and energy transport, and vitamin deficiency in children with ASD than controls, plasma 25(OH)D did not differ across study groups [116]. The lack of difference could in part be attributed to some methodological issues; neither cases nor controls were assessed for autism manifestations by researchers and confounders such as ethnicity, season of blood sampling, medical conditions and medication use were not controlled for in statistical analysis. Approximately half of the cases were on different medications including psychopharmaceuticals, central nerve system stimulants, anticonvulsants, gastrointestinal medication, asthma/allergies and insulin, and 11% of controls on either anti-inflammatory or anti-incontinence medications. 

The study by Ugur *et al.* (2014) [79] was conducted in Turkey, where a national free vitamin D supplementation campaign has been implemented by government to eliminate vitamin D deficiency rickets [122]. The comparable levels of 25(OH)D among ASD cases and controls in this study could therefore be attributed to the nationwide vitamin D supplementation after initiation of ASD. 

The cause and effect relationship from these case-control studies cannot be determined. Patients with ASD are likely to have medical conditions and use medications—affecting vitamin D metabolism or absorption—that have not been considered in these studies. Inflammation [120,121], gastrointestinal issues [123,124], antiepileptic drugs [125] and genetics are among these factors. Furthermore, children with ASD may receive less sunlight due to lower outdoor activity and have inadequate intake of vitamin D [114,118,126,127,128,129,130,131,132,133,134,135,136], *i.e.*, reverse causality. 

Along those lines, according to parental report, ASD children had significantly more sedentary behaviours than typically developing controls, a finding confirmed by Neumeyer *et al.* (2013) and Bener *et al.* (2014) [118]. Approximately 11% and 73% of ASD children and typically developing children had high physical activity levels, respectively [114], and fewer ASD children were exposed to sun than healthy controls (*p* = 0.002) [118]. 

Moreover, due to repetitive and restricted dietary behaviours, ASD children are at increased risk of low dietary vitamin D intake [128,129]. Evidence suggests that a large proportion of children with ASD do not meet vitamin D requirements [126,132,133,134,135,136]. Using three-day food records, Stewart *et al.* (2015) reported a probable risk of inadequate intake of six micronutrients in >40% of children with ASD, one of which was vitamin D [126]. Even though vitamin D intake was improved in response to vitamin D supplementation, it did not reach the adequate intake in 30%–40% of children. The scenario would be worse if more children of African American, mixed-race/ethnicity and less educated parents were enrolled because the socio-demographic characteristics of the study population was biased toward highly educated parents and those of white ethnicity who might have been more concerned about nutrition. Average vitamin D intake was 62% and 49% of dietary recommended intake (DRI) in boys with ASD aged 7–8 and 9–12 years, respectively [136]. Although these studies illustrated a large proportion of children with ASD not meeting the recommendations for vitamin D intake, they did not find any evidence that dietary intake of vitamin D in children with ASD differed from that of typically developing controls.

It has been argued that low dietary intake of vitamin D is a public health issue, and is not specific to children with ASD [131,137,138]. In a recent case-control study, Mari-Bausent *et al.* (2015) reported children with ASD having lower dietary vitamin D intake than healthy controls, though the difference was not statistically significant (79 ± 60 *vs.* 104 ± 97 IU/day, respectively, *p* = 0.06) [131]. A large proportion of both groups did not meet the dietary recommended intake for vitamin D (88% of children with ASD and 75% of typically developing children, *p* = 0.16), a finding confirmed by others [137,138].

However, several case-control studies have shown that children with ASD have lower vitamin D intakes than typically developing controls [114,128,129,130]. Emond *et al.* (2010) and Zimmer *et al.* (2012) reported that children with ASD had late introduction of solids after six months, were difficult to feed and very choosy from 15–54 months, had less varied diet from 24 months, and ate fewer food on average at older ages, all of which affected their nutritional status [128,129]. Both studies showed that children with ASD had significantly lower dietary intakes of vitamin D than controls. Daily vitamin D intake was 199 ± 176 IU and 320 ± 119 IU in ASD and typically developing children, respectively (*p* < 0.05) [129]. More children with ASD did not also meet the estimated average requirement (EAR) for vitamin D intake than healthy controls (79% *vs.* 55%, *p* = 0.01) [130], and more selective eaters with ASD were at increased risk for inadequate intake (58% *vs.* 5%, *p* = 0.01) [129]. Thus, low vitamin D status could be a risk factor for ASD if it occurs during critical period of development, or alternatively, could be a condition that appears as the disease progresses.

### 3.3. Vitamin D-ASD-Related Areas Providing an Indication for Secondary Prevention

Here, we will review the literature regarding a vitamin D-related measure—that is the treatment of low vitamin D status when ASD has already occurred—which can be considered as a disease modifier, and may reduce the symptoms of ASD in patients. 

#### Vitamin D Intervention to Treat ASD 

We included all studies reporting using vitamin D supplements among populations with ASD. We excluded those studies using vitamin D containing multivitamins or minerals only because those doses are relatively low and the effect of vitamin D *per se* cannot be determined from these interventions. 

Our literature search identified six studies reporting on the effect of vitamin D supplementation on autism symptoms (Table 4); one clinical quality assurance practice [119], one case report [139], three open label intervention trials [111,117,141], and one randomised controlled trial [140]. The full text for one article was not available at the time of writing this review, as such the critical appraisal could not be employed and the data presented here are based on the abstract [140]. We found no randomised placebo controlled trials, but three ongoing studies from Egypt (NCT02550912), Ireland (NCT02508922) and New Zealand (ACTRN12615000144516). Four studies were among children with ASD [111,117,139,140], one among developmentally delayed children with and without ASD [141], and one in adult patients with ASD and other psychiatric disorders [119]. Humble *et al.* (2010), in a clinical quality assurance project, reported a considerable improvement in psychosis or depression with the effective treatment of vitamin D deficiency in several patients. In their clinical practice they used 1600–4000 IU vitamin D_3_ daily or 35,000–70,000 IU vitamin D_2_ once weekly [119]. Autistic and schizophrenic patients had the lowest median 25(OH)D concentrations compared to other psychiatric disorders, 32 (25th–75th percentile, 23–39) nmol/L and 35 (24–53) nmol/L, respectively, *vs.* >40 in other patients. No conclusion can be drawn from this study because it was not designed to assess the efficacy of vitamin D on autism and it was part of clinical practice, no placebo or control arm was included, different doses and types of vitamin D was used, and levels of 25(OH)D after treatment was not reported.

In a recent case report, Jia *et al.* (2015) reported that shifting serum 25(OH)D concentration in a 32 month old child with ASD from 31 nmol/L to 203 nmol/L after two months of high dose vitamin D_3_ supplementation (150,000 IU per month administered intramuscularly plus 400 IU per day orally) improved autistic core symptoms [139]. Scores on Autism Behaviour Checklist, Childhood Autism Rating Scale (CARS) and Severity of Illness of Clinical Global Impression increased from 80, 35 and 6 at the baseline to 39, 28 and 4 at the follow up, respectively. The findings from this single patient cannot be generalised to all autistic patients but it does encourage further research in this area. Following on from this case report, Feng and colleagues (2016) assigned 37 ASD children from a pool of 215 children with ASD (25(OH)D <75 nmol/L) to receive the same dose of vitamin D supplementation for three months [117]. The authors reported a significant improvement in scores on CARS (total) and Autism Behaviour Checklist (total and all subscales apart from sensory scores), which was more pronounced in 3 year old or younger children than older children.

Saad *et al.* (2015), in an open label trial, assigned 106 children with ASD and 25(OH)D concentrations <75 nmol/L to receive daily 300 IU/Kg not exceeding daily 5000 IU for three months. This study was part of a case-control study investigating differences in 25(OH)D concentrations among autistic children and healthy age and sex matched controls (previously discussed). Symptoms of ASD were measured using CARS and Aberrant Behaviour Checklist (ABC). CARS scores decreased by 3.5–6.5 points in all 16 patients with final 25(OH)D >100 nmol/L, by 1.5–4.5 points in 31 out of 49 patients with 25(OH)D levels 75–97.5 nmol/L and no improvements in all 18 patients with 25(OH)D levels <75 nmol/L had improvements. With the exception of inappropriate speech subscale of ABC, all other measures (irritability, stereotypic behaviour, social withdrawal and hyperactivity) improved significantly after treatment. This study had a relatively large sample size and also limited seasonal effects by drawing all blood samples during the summer months, though the baseline and follow-up levels of 25(OH)D were not reported. Other limitations of this study were its drop-out rate of approximately 22% and the lack of a placebo or comparison arm. 

In another study from Turkey, however, despite improvement in scores on ABC and Denver II in both treatment and control groups, Ucuz *et al.* (2015) failed to show any significant differences across groups [141]. To note, the improvement was more pronounced in those receiving vitamin D supplement. The authors recruited children with developmental delay with and without ASD, divided them into two groups; those with 25(OH)D concentration <50 nmol/L who received daily 5000 IU vitamin D supplement for one month and daily 400 IU vitamin D for further two months if 25(OH)D was not corrected at one month follow up, and ≥50 nmol/L who did not receive vitamin D supplements, and performed subcategory analysis for children with developmental delay with ASD and without ASD. This study had a small sample size of <11 participants per group. Furthermore, children were not randomly assigned to study groups and neither baseline nor follow up 25(OH)D concentrations were reported. It should be noted that the findings from this study cannot be extrapolated to children with ASD only. The brain chemistry of children with ASD may differ from that of children with developmental delay [142], and as such the response to vitamin D supplement—that may affect brain chemistry [143]—could vary. 

In a randomised controlled trial including only children with ASD, Azzam *et al.* (2015), similarly, reported comparable improvements in scores on CARS, social IQ, and Autism Treatment Evaluation Checklist in both vitamin D supplemented (six months) and control groups [140]. Although no quality appraisal can be performed for this study, what is clear is that this study had a small sample size and lacked a placebo arm. In summary, although these early interventional studies provided encouraging results, no firm conclusions can be drawn until randomised placebo-controlled trials with sufficient sample size are undertaken.

## 4. Mechanistic Pathways

Vitamin D receptors (VDR) and enzymes involved in vitamin D metabolism have been identified in several regions of the brain including neurons and glial cells [144]. VDR has been shown to be present early in development, to increase during development, and to be present in the adult brain—all indicative of a role for vitamin D in the developing and adult brain [145]. Low vitamin D status has been implicated in pathophysiology of ASD in several ways. It has been hypothesised that ASD is a combination of both organ specific physiologic and systematic abnormalities such as *de novo* gene mutations, oxidative stress, impaired detoxification system, inflammation, immune dysregulation, abnormal neurotrophic factor and neurotransmitter levels, and seizures, at least in a subset of individuals with ASD [8]. Mounting evidence suggests that low vitamin D status is involved in the aetiology of the mentioned abnormalities [105].

Autism is considered to be driven genetically [146,147], though only small percentage of cases are clearly linked to genetic causes [22]. The only study linking vitamin D metabolic gene variants to ASD risk is by Schmidt *et al.* [148]. The authors illustrated that the risk for ASD was increased in children inheriting the AA genotype of the GC gene (D binding protein), the GG genotype of the CYP2R1 gene (a catalyst enzyme involved in the transformation of vitamin D to 25(OH)D), and paternal *Taql* and *Bsml* genotypes of the VDR gene, highlighting the possible aetiological role of low vitamin D in ASD.All of these results support an association between vitamin D status and ASD.

Vitamin D regulates cell profilation and differentiation and can protect the genome from daily life insults such as oxidative stress and toxins (for a comprehensive review refer to [149]). Treatment with vitamin D has been shown to decrease 8—hydroxy–2—deoxyguanosin, a marker of endogenous oxidative damage to DNA [150], increase Bax, a protein coding gene involved in apoptosis [150], regulates poly-ADP-ribose polymerase activity in the DNA damage response pathway [151], stabilize chromosomal structure and prevent double strand breaks [152].

There are presently several lines of evidence indicating oxidative stress and mitochondrial dysfunction are prevalent in individuals with ASD [3,10,11,12,13,14,15,16]. Recent evidence shows that children with ASD have lower levels of total glutathion and glutathione peroxidase (an enzyme involved in antioxidant defense and detoxification), higher levels of oxidised glutathione and F2—isoprostane (a marker of oxidative stress and lipid peroxidation) and reduced capacity for methylation than typically developing children [16,153]. Oxidative stress and reduced redox/antioxidant capacity in cerebellum and temporal cortex have been shown to have functional consequences for chronic inflammatory response, mitochondrial dysfunction and protein and DNA damage [3], and in peripheral tissue have been associated with anti-neuronal positivity [16] and clinical features of autism [15]. Evidence shows that 25(OH)D concentrations correlate significantly positively with glutathione levels in healthy adult populations [154], and low vitamin D status is associated with increased inflammatory, oxidative and endothelial activation biomarkers in obese individuals [155]. It has been documented that treatment with vitamin D has a protective effect on persistent biochemical features comparable to those reported clinically in autistic patients and ameliorates neurotoxicity, inflammation and DNA damage in propionic acid-intoxicated rats [156].

Exposure to heavy metals and impaired detoxification system, another abnormality in a subset of individuals with ASD, have also been involved in the aeitiology of ASD [157]. Alabdali *et al.* (2014) reported autistic children having higher levels of mercury and lead than healthy controls, and lower levels of glutathione-transferase and vitamin E in plasma [157]. Using cell culture models, Shinpo *et al.* (2000) and Garcion *et al.* (1999) indicated that pretreatment of cells exposed to toxicants with 1,25(OH)_2_D attenuated reactive oxygen species and nitric oxide, and enhanced intracellular gamma-glytamyl transpeptidase (an enzyme involved in glutathione synthesis) and glutathione in the intracellular pool [158,159].

Furthermore, evidence suggests that children with ASD have elevated levels of pro-inflammatory cytokines including, interleukin 6 (IL–6), tumour necrosis factor alpha (TNF–α), and interferon gamma (IFN-γ) in different tissues [3,4,5,6,7]. The activation of microglia, the unique resident cells of the central nervous system, is the prominent feature of autism [160] and is associated with increased several pro-inflammatory cytokines [161]. It is well known that vitamin D has an effect on immune system and can directly affect immune cells. For example, vitamin D metabolites have been shown to decrease the secretion of IL-6 and TNF-α [162], enhance the expression of anti-inflammatory cytokines such as interleukin 10 (IL–10) from activated *B* cells [163], and to direct dendritic cells (DCs) toward a more tolerogenic state [164]. 

Autism is also considered as an autoimmune disease in a subset of individuals. Elevated autoantibodies in the brain, specifically anti-myelin basic protein antibodies have been reported in autism [9]. Mostafa and Al-Ayadi reported significantly higher proportion of children with ASD having anti-MAG antiautoantibodies, anti-ganglioside M1, anti-neuronal and anti-nuclear antibodies than typically developing controls, that positively correlated with the severity of autism symptoms (CARS scores) [109,165,166,167]. Family history of autoimmune diseases was reported to be higher in children with ASD than controls [166,168]. Using a genome-wide data set of ASD and autoimmune disorders, Multiple Sclerosis (MS) was identified to be significantly associated with ASD [169]. Both conditions have been characterised by immunological abnormalities and dysfunctional myelination, at least in a subset of individuals [170,171]. Furthermore, anti-MAG antiautoantibodies correlated negatively with serum 25(OH)D concentrations (*r* = −0.86, *p* < 0.001) [109], and anti-nuclear antibody positive healthy controls were significantly more likely to be deficient in vitamin D than antibody negative healthy controls (71% *vs.* 22%, respectively, *p* = 0.003) [172]. Vitamin D has also been shown to modify the expression of several genes involved in axogenesis and myelination [173]. These findings suggest an important role of vitamin D in autoantibody production and ASD pathogenesis, perhaps similar to other autoimmune diseases, such as MS and systemic lupus erythematosus [172,174]. 

Evidence suggests that vitamin D has a neuroprotectory effect through its neurotrophic and immunomodulatory properties. Neurotrophins are involved in the development, maintenance, survival and synapsis of neurons [105]. Abnormal levels of several neurotrophic factors such as nerve growth factor (NGF) and brain-derived neurotrophic factors (BDNF) have been reported in subset of individuals with ASD [175,176]. Vitamin D analogues have been shown to upregulate NGF in cultured glial cell and embryonic hippocampal cells [177,178], to decrease the percentage of cultured hippocampal cells undergoing mitosis [178], and to decrease BDNF and concommitantly to improve memory in postmenopausal women [179]. 

Multiple lines of evidence suggest an involvement of dysregulated neurotransmitter systems (serotonergic, oxytocinergic and dopaminergic systems) in autism. These systems play key roles in neurotransmission, brain maturation, cortical organisation and behaviours (including social behaviour and repetitive behaviour) [180,181,182,183,184]. Lower levels of plasma oxytocin [185] and abnormal serotonin concentrations in the brain and tissues outside the blood-brain barrier have been reported in populations with ASD [186,187]. While the binding of brain serotonin transporter was significantly lower in adults with high functioning autism than healthy controls, the binding of brain dopamine transporter was significantly higher in autistic patients [184]. Recently, two ASD associated independent gene variants, including a gene variants regulating STX1 phosphorylation (regulates neurotransmitter transporter) and dopamine transporter, that alter dopamine transporter function have been identified [188]. Vitamin D receptors have been identified in dopamine neurones in humans substantia nigra [145], and vitamin D response elements on genes involved in serotonin and oxytocin synthesis [189]. Pre-treatment with calcitriol has been shown to protect rats’ striatum and accumbens from methamphetamine-induced reduction in dopamine and serotonin [143]. 

Finally, the above mentioned disorders may contribute to the aetiology of epilepsy, which is highly prevalent in populations with ASD [190]. According to retrospective follow up studies, 37% and 25% of the individuals with autism exhibited epilepsy and epileptic seizures, respectively [191,192]. Infants of mothers taking antiepileptic drugs during pregnancy have been shown to be at increased risk of being diagnosed with ASD and having low 25(OH)D concentrations [193]. Vitamin D and its analogues have been shown to increase seizure threshold, potentiate anticonvulsant activity of antiepileptic drugs, and to decrease the severity of seizures in animal studies [194,195,196]. Correction of vitamin D deficiency in patients with seizures, decreased the frequency of seizures by 30%–40% [197,198]. Vitamin D exerts its roles, in part, through its antioxidant and anti-inflammatory effects and also through its inhibitory effect on Ca^2+^ influx in the brain. Calcitriol upregulates the expression of calcium binding proteins (calbindin and parvalbumin) and inhibits the expression of L-type Ca^2+^ channels [199]. It should be noted that at the same time, antiepileptic drugs impair vitamin D and calcium metabolism, leading to reduced levels of vitamin D and calcium [125], and these deficiencies can, on the other hand, exacerbate the condition. 

## 5. Summary

Low vitamin D status in utero, postnatal and in early childhood has been hypothesised as a risk factor for neurodevelopmental disorders, specifically ASD. Animal and human cellular, biological, and physiologic studies have provided compelling evidence for numerous roles of vitamin D in various body processes, some of which are involved in the pathobiology of ASD. Our literature review identified a large number of observational studies but very few intervention trials investigating the relationship between vitamin D and ASD. Conclusions are not yet possible due to the inconsistent results, different methodological approaches employed, and very few trials in the current literature. However, there are some indications that early exposure to inadequate vitamin D may interact with other factors and contribute to the aetiology of autism, low vitamin D status might be highly prevalent in populations with ASD, and intervention with vitamin D might be beneficial in reducing autism symptoms among those who have ASD. Therefore, there is an urgent need for randomised controlled trials of vitamin D in populations genetically predisposed to ASD and in populations with ASD to confirm these findings and to generate evidence-based clinical recommendations for the prevention of ASD and management of ASD symptoms. Until better data are available, health care providers and researchers are advised to consider vitamin D-related factors as potential preventive and disease-modifying measures for ASD. 

## Figures and Tables

**Table 1:** Risk of Autistic Disorder (AD) in relation to latitude & Risk of Pervasive Developmental Disorder (PDD) in relation to latitude.

Reference	Country, Area	Latitude	Age	Diagnosis	Prevalence/10,000	CI
Risk of Autistic Disorder (AD) in relation to latitude						
[36]	China, Tianjin	39.13°N	8	DSM-IV/CABS-CV, CARS-CV	10.9	3.4–25.4
[37]	UK, South East Thames	51.24°N	7	ICD-10/CHAT, CR, PDD-Q	30.8	22.9–40.6
[38]	UK, Staffordshire and Cannock	52.80°N	4–6	ICD-10, DSM-IV/multidisciplinary screening ADI-R	18.9	14.1–25.0
[17]	UK, Staffordshire	52.80°N	2.5–6.5	DSM-IV/Clinical evaluation, ADI-R	16.8	11.0–24.6
[39]	Sweden, Goteborg	57.70°N	3–6	ICD-10/Clinical evaluation, ADI-R	46.4	16.1–76.6
[40]	Sweden, Karlstad	59.37°N	7	ICD-10/ASSQ (cut off teachers only: 17), Clinical evaluation, ADI-R	60.0	19.0–141.0
[41]	Finland, Northern Ostrobothnia	63.91°N	8	DSM-IV-TR, DSM-5/ASSQ (cut off: 30 parents and teacher combined), ADI-R, ADOS-3	41.0	26.0–64.0
Risk of Pervasive Developmental Disorder (PDD) in relation to latitude						
[42]	UAE, 3 regions	24.46°N	3	DSM-IV/ASC (cut off: 15), Clinical evaluation	29.0	0–79.0
[43]	Iran, Country wide	32.00°N	5	DSM-IV/National screening, SCQ, ADI-R	6.3	5.8–6.7
[44]	Argentina, San Isidro	34.28°S	0–5	DSM-IV/PRUNAPE, BDI, VABS, multi-disciplinary evaluation	13.1	-
[45]	Japan, Toyota	35.08°N	5–8	DSM-IV/Integral screening system, Direct clinical evaluation	181.1	-
[37]	UK, South East Thames	51.24°N	7	ICD-10/CHAT, CR, PDD-Q	57.9	46.8–70.9
[38]	UK, Staffordshire and Cannock	52.80°N	4–6	ICD-10, DSM-IV/multidisciplinary screening, ADI-R	58.7	45.2–74.9
[17]	UK, Staffordshire	52.80°N	2.5–6.5	DSM-IV/Clinical evaluation, ADI-R	61.9	50.2–75.6

DSM-IV, Diagnostic and Statistical Manual of Mental Disorders-Fourth Edition; CABS-CV, Clancy Autism Behaviour Checklist-Chinese Version; CARS, Childhood Autism Rating Scale; ICD-10, International Classification of Diseases and Related Health Problems (10th edition); CHAT, Checklist for Autism in Toddlers; CR, Checklist for referral; PDD-Q, Pervasive Developmental Disorder-Questionnaire; ADI-R, Autism Diagnostic Interview-Revised; ASSQ, Autism Spectrum Screening Questionnaire; ADOS-3, Autism Diagnostic Observation Schedule-Module 3; ASC, Autism Screening Questionnaire; SCQ, Social Communication Questionnaire; PRUNAPE, Prueba Nacional de Pesquisa; BDI, Battelle Developmental Inventory; VABS, Vineland Adaptive Behaviour Scales.

**Table 2:** Risk of autism in mothers born outside the reference country and according to mothers’ ethnicity.

Reference	Reference Country/Year	Cohort	Age (Year)	Cases Number	Cases Ascertainment	Mother’s Country of Birth/Ethnicity	Odds Ratio (95% CI)	Covariates
[64]	Sweden 2002	1987–1994	<10	408	AD/ICD 9	Europe and North America	1.1 (0.5–2.5)	Maternal age, parity, smoking during pregnancy, hypertensive disease, diabetes, pregnancy bleeding, mode of delivery, season of birth, gestational age, birth weight, Apgar score, congenital malformation
						Outside Europe and North America	3.0 (1.7–5.2)	
[68]	US/California 2002	1989–1994	-	4381	AD/DSM-III-R or DSM-IV	Other US states	0.9 (0.8–1.0)	Gender, birth weight, plurality, birth order, maternal age, maternal race, maternal education RR
						Mexico	0.6 (0.5–0.7)	
						Other	1.1 (1.0–1.2)	
[65]	Denmark 2005	1984–1998	<10	818	AD, atypical autism/medical record registry/ICD-10	Scandinavia and Europe	1.0 (0.8–1.4)	Age, sex, interaction between age and sex, calendar year, history of autism or broader autism in siblings psychiatric disorders RR
						Outside Europe	1.4 (1.1–1.8)	
[62]	Denmark 2006	1990–1999	<10	473	AD/ICD-8 and 10	Foreign Citizenship (not mentioned)	1.7 (1.3–2.4)	Maternal and parental age, maternal citizenship, birth weight, gestational age, Apgar score, irregular foetal position, congenital malformation, psychoactive medicine use in pregnancy
[66]	Australia 2008	1990–1996	<5	368	ASD/Surveillance/DSM-IV	Outside Australia	1.4 (1.0–1.9)	Gender, maternal age ≥35, gestational age <37
[67]	Sweden 2010	1980–2005	<25	250	AD and AS/DSM-IV or DSM-III or ICD-10/ADOS-G and ADI-R	Outside Nordic countries:		Year of birth, maternal age ≥40, gestational age <37, gestational age-adjusted birth weight
						AD	2.2 (1.6–3.1)	
						AS	0.6 (0.3–1.0)	
						Sub-Saharan Africa	5.6 (2.9–10.6)	
						South or Central America	3.1 (1.3–7.1)	
						East Asia	2.9 (1.4- 6.1)	
						Western Europe/USA	2.8 (1.2–6.5)	
						Previous Eastern Europe	2.1 (1.3–3.3)	
						Middle East/North Africa	2.0 (1.2–3.2)	
[63]	UK 2010	1999–2005	<18	428 (267, Wandsworth and 161 in Lambeth)	ASD/2 boroughs/Multidisciplinary team assessment/ICD-10 using ADI-R, DISCO, ADOS	Other European		Family size RR
						L	1.3 (0.6–2.8)	
						W	1.2 (0.8–1.9)	
						African		
						L	7.9 (5.4–11.6)	
						W	3.3 (2.4–4.5)	
						Caribbean		
						L	10.0 (5.5–18.1)	
						W	8.9 (5.9–15.5)	
						Asian		
						L	4.0 (2.0–7.8)	
						W	2.1 (1.3–3.3)	
[61]	Sweden 2012	2001–2007	0–17	3918	ASD/Medical registry/Multidisciplinary teams/DSM-IV	African, American, Asian and European		Maternal and parental age, family disposable income, for subsample, birth weight, gestational age, Apgar score at 5 min after birth
						Low functioning	1.2 (1.0–1.4)	
						High functioning	0.5 (0.4–0.6)	
[70]	Netherlands 2013	1998–2007	-	518	ASD/Psychiatric case registry/DSM-IV	Developing countries		Gender and paternal age RR
						ASD	0.6 (0.5–0.9)	
						AD	1.4 (0.9–2.4)	
						AS and PDD-NOS	0.4 (0.3–0.6)	
						Developed countries		
						ASD	0.9 (0.6–1.3)	
						AD	1.6 (0.8–3.5)	
						AS and PDD-NOS	0.6 (0.4–1.1)	
[69]	US/Los Angeles 2014	1995–2006	-	7540	AD/DSM-III-R/ICD-9-CM/ADOS	White foreign	1.0 (0.9–1.2)	Maternal age, gender, birth year, birth type, parity, gestational age, birth weight, pregnancy complications, trimester pregnancy care began, maternal education, insurance and diagnostic variability (regional centres) RR
						Black foreign	1.8 (1.4–2.2)	
						Mexico	1.1 (1.0–1.2)	
						Central/South America	1.3 (0.9–1.1)	
						China	0.7 (0.6–0.8)	
						Japan	0.7 (0.5–1.0)	
						Korea	1.0 (0.8–1.2)	
						Philippines	1.3 (1.1–1.4)	
						Vietnam	1.4 (1.2–1.7)	

AD, Autistic Disorder; ICD, International Classification of Diseases; US, The United States; DSM, Diagnostic and Statistical Manual of Mental Disorders; ASD, Autism Spectrum Disorders; ADOS, Autism Diagnostic Observation Schedule; ADI-R, Autism Diagnostic Interview-Revised; AS, Asperger; UK, The United Kingdom; DISCO, Diagnostic Interview for Social and Communication Disorders.

**Table 3:** Risk of autism according to seasonality of conception and birth.

Reference	Country/Year	Diagnosis	Case-Control Numbers	Case—Control Characteristics	Confounders/Covariates	Excess Conception	Excess Birth
[92]	Canada 1986	DSM-III	179-NR	Low functioning (IQ < 50) and high functioning (IQ > 50) autism, non-verbal and verbal autism from medical records from two different centres-live births			March *vs.* Sep-Feb
							Spring-early summer *vs.* winter and autumn (Aggregated sample)
							Spring *vs.* winter and autumn (Low functioning, nonverbal, male)
[91]	Japan 1988	DSM-III	80–71,013	Native infantile autism <8 years from clinic outpatients-children <8 years from annual reports			Second quarter of the year (corresponding to spring) *vs.* first and third quarter*
[90]	Sweden 1990	DSM-III-R	100-NR	Cryptogenic autism-populations born in Sweden (Central Bureau of statistics)	Cases with medical conditions and of mothers immigrated to Sweden from non-European countries were excluded		March
[89]	UK 1992	ICD-9, DSM-III	1435–196–121–24,957,169	National autistic sample-clinic sample-sibling controls-live births			Significantly deviated from the general populations’ expected moth of birth (national sample)
							December, January, June, July and October
[88]	Denmark 1994	ICD-9	328-NR	Infantile autism-autism like disorder-borderline psychosis from clinic outpatients-live births			March and April *vs.* November December
[87]	Israel 1995	DSM-III-R	188–1,992,410	Infantile autism-live births			March and August
[86]	International 1999	DSM-IV, ICD-10	620–284	Cases with autism from international multisite field trial for DSM-IV-Individuals with mental retardation from patients of a clinic			No association
[84]	Netherlands 2000	ICD-9	1031-NR	National registry of mentally retarded patients with AD and PDD-NOS (IQ < 35)-general population birth data			No association (month and season) (Aggregated sample)
							Second quarter of the year (Low functioning)
[85]	US 2000	DSM-III-R	175–123	High and low functioning autism (verbal IQ cut off of 65) recruited for a research project-full siblings and half siblings	Arbitrary assignations of month to season		No association (aggregated sample)
							March (more low functioning and socially passive autism) (Boston subset)
[64]	Sweden 2002	ICD-9	408–2040	Infantile autism <10 years from medical birth register-birth register	Maternal age, parity, smoking, mother’s country of birth, hypertensive disease, diabetes, pregnancy bleeding, mode of delivery, gestational age, birth weight, Apgar score, congenital malformation		No association
[83]	Israel 2006	ICD-10	211–311,169	ASD adolescents (age of 17) from military medical registry-live births	Year of birth, socioeconomic status		No association
[77]	Denmark 2007	ICD-10	1860–407,117	ASD and ASD subcategories from psychiatric registry-live births	General trend for increase in incidence over time, follow up time, length of gestation	No association	No association
[82]	US 2008	DSM-IV	1051–1,458,011	ASD singletons and multiple births from medical records-statistics data for singleton and multiple live births	Number of births and gender		Spring (April), summer (late July) and autumn (October) *vs.* winter (December and January) (Singletons and multiple births)
[80]	Denmark 2010	ICD-10	317–733,826	Infantile autism from medical birth register-live births	Gender, maternal smoking status, irregular fetal presentation, birth weight, gestational age, Apgar score, parental age, maternal citizenship, congenital malformation		Winter (October to March) *vs.* Summer (April to September), 2.21 (1.24–3.94) *vs.* 1.02 (0.41–2.50)
[81]	Egypt2010	DSM-IV	70–40	ASD (recruited for the purpose of the study)-non ASD healthy controls			No significant difference
							June (26.7%) followed by March and April (11.4%,)
[76]	UK 2010	ICD-10	86–13,892	ASD from medical and educational records-live births		Summer *vs.* winter 2.08 (1.18-3.70)	Spring *vs.* autumn (reference), 1.86 (1.01-3.37)
[75]	US 2011	ICD-9	19,328–6,585,737	Full syndrome autism <6 years and live births <6 years from dataset	Gender, race/ethnicity, Preterm birth, maternal age, maternal education, maternal place of residence at childbirth and maternal year of conception	Winter (January, February and March) *vs.* Summer, 1.06 (1.02–1.10)	November *vs.* April (reference), 1.12 (1.05–1.20)
[74]	US 2012	DSM-IV	8,074–3,888,495	AD not comorbid with mental retardation-live births	Gender, parental age and education, race and ethnicity, insurance status, preterm birth and low birth weight	Winter (the last 3 weeks of November and first week of December), 2.11, 1.72 and 1.53 in 1994, 1995 and 1996, respectively	
[79]	Turkey 2014	DSM-IV	54–54	ASD (recruited for the purpose of the study)-non ASD healthy controls			No association
[78]	Sweden 2015	DSM-IV-TR	58–58	ASD (recruited for the purpose of the study)-non ASD siblings			No association in children of Middle Eastern/African ethnicity
							Spring *vs.* Summer, 38% *vs.* 10% in ASD and 18% *vs.* 35% in non ASD in children of Sweden and European ethnicity

**Table 4:** Vitamin D interventions to prevent or treat Autism Spectrum Disorder (ASD).

Reference	Country/Year	Study Design/Country	Population Characteristics	Intervention	Baseline 25(OH)D (nmol/L)	Follow up 25(OH)D (nmol/L)	Outcome Measure
**Vitamin D intervention to prevent ASD**							
[106]	US 2016	Prospective open label intervention trial	Pregnant mothers of autistic children	Daily 5000 IU during pregnancy and 7000 IU during breastfeeding	All but two >50	70–198	Recurrence rate of autism in new born siblings was 5% which was lower than that reported in the literature (20%)
				Daily 1000 IU during the first three years of life if child was not breastfed			
**Vitamin D intervention to treat ASD**							
[119]	Sweden 2010	Clinical quality assurance project	Autism and other psychiatric disorders (mean age of 43.7 years)	Daily 1600–4000 IU vitamin D_3_ or 35,000–70,000 IU vitamin D_2_ once weekly	31.5 (23–39) in autistic patients and 45 (31–60) in all patients *	-	Improvement in psychosis or depression
[139]	China 2015	Case study	A 32-month old male toddler	Monthly 150 000	31.3 **	203	ABC ^1^ (from 80 to 39)
				IU IM and			CARS (from 35 to 28)
				daily 400 IU orally			Severity of Illness of Clinical Global Impression (from 6 to 4).
				for two months			
[111]	Egypt 2015	Open label intervention trial	106 autistic children with 25(OH)D <75 nmol/L	Daily 300 IU vitamin D_3_/Kg not exceeding 5000 IU/day for three months	<75	-	ABC^2^: Improvements in irritability (0.02), hyperactivity (0.03), social withdrawal (0.01) and stereotypic behaviour (0.04)
							No improvements in inappropriate speech
							CARS: Improvements in total (*p* < 0.001), relating to people (*p* < 0.001), imitation (*p* < 0.001), body use (*p* = 0.01), object use (*p* = 0.01), adaptation to change (*p* = 0.004), listening response (*p* = 0.01), visual response (*p* = 0.003) and general impression (*p* < 0.001)
							No improvements in fear, verbal communication, activity level, nonverbal communication and intellectual response
							The improvement was more pronounced in those with final 25(OH)D >100 nmol/L
[140] ^1^	Egypt 2015	Randomised controlled trial	21 autistic children assigned to vitamin D or no treatment groups	-	-	-	CARS, social IQ and ATEC: Improved in both groups
							Improvement was not significantly different across groups
[141] ^2^	Turkey 2015	Open label intervention trial	Toddlers with developmental delay without and with ASD ^2^ (2–5 years old), and 25(OH)D <50 nmol/L (*n* = 11, cases) and ≥50 nmol/L (*n* = 10, controls)	Baseline 25(OH)D <37.5 nmol/L: daily 5000IU for one month and then daily 400IU for two months if 25(OH)D is between 37.5 and 50 nmol/L after one month	-	-	ABC ^1^ and Denver II: Significant improvement in both groups (ABC, from 90 ± 19 to 59 ± 15 in cases and from 77 ± 22 to 64 ± 29 in controls; Denver II, from 64 ± 13 to 72 ± 17 in cases and from 73 ± 11 to 80 ± 12 in controls).
				Baseline 25(OH)D between 37.5 and 50 nmol/L: daily 400 IU for one to three months depending on the level at one month			
							Neither baseline nor endpoint scores were significantly different across groups, but improvement was more pronounced in cases
[117]	China 2016	Open label intervention trial	37 autistic children with 25(OH)D <75 nmol/L	Monthly 150,000 IU IM and daily 400 IU orally for three months	-	-	CARS: Improvement in total scores
							ABC ^1^: Improvement in total and social skills, body and object use, language, and social and self-help
							Improvement was more pronounced in younger children (≤3 *vs.* >3 years old).

* Median (25th–75th percentile); ** The values were ng/mL, they have been converted to nmol/L for easy comparison (1 ng/mL = 2.5 nmol/L); ^1^ The full text was not available at the time of writing this manuscript and are information provided here are based on the abstract; ^2^ The data for children with developmental delay and ASD are reported here; ASD, Autism Spectrum Disorder; 25(OH)D, 25 hydroxyvitamin D; IU, international unit; D_3_, cholecalciferol; D_2_, ergocalciferol; ABC^1^, Autism Behaviour Checklist; CARS, Childhood Autism Rating Scale; ABC^2^, Aberrant Behaviour Checklist; ATEC, Autism Treatment Evaluation Checklist; Denver II, Denver Developmental Screening Test II.

**Table 5:** Vitamin D status in ASD patients-Case-control studies.

Reference	Country/Year	Case-Control Characteristics	Assessment Tools	Covariates/Confounders	Boys: Girls	*p*-Value	25(OH)D Concentration (nmol/L )*	
							Cases	Controls
[81]	Egypt 2010	70 children with autism (mean age of 5.3 ± 2.8)	DSM-IV	Season of birth	-	<0.001	71.3 ± 41.0 **	100.3 ± 29.5
		40 age matched healthy controls of the same socioeconomic status controls (thoroughly examined)						
[113]	US 2011	71 Caucasian males with ASD (4–8 years old)	DSM-IV/ADOS	Covariates: age, BMI, use of supplement, antiepileptic medication, season of enrolment	Only male	n.s.	49.8 (range, 27.0–77.5) **	42.5 (range, 19.5–70.8)
							(CFD)	
						n.s	49.0 (range, 22.3–76.8)	
		69 age matched typically developing controls					(NCFD)	
[116]	US 2011	55 children with ASD (aged 5–16 years old)	Diagnostic confirmation from pediatrician or other professionals/PDD-BI (autism composite), ATEC, SAS		49: 6	n.s.	74.6 ± 21.0 **	71.5 ± 21.0
		44 age, sex and geographically similar distribution						
[110]	Brazil 2012	24 children with ASD (mean age of 7.4 ± 2.7 years)	DSM-IV		18:6	<0.001	66.2 ± 8.7 **	101.3 ± 7.8
		24 age and sex matched healthy controls						
[109]	Saudi Arabia 2012	50 children with ASD (5–12 years)	DSM-IV/CARS (severity)	All blood samples drawn in summer	39:11	<0.001	46.3 (IQR, 35.0) **	82.5 (IQR, 27.5)
		30 age and sex matched healthy controls						
[114]	US 2012	18 boys with ASD (mean age of 10.6 ± 0.4 years)	DSM-IV/ADOS	Season of blood collection, bone age	NA	0.06	66.8 ± 4.8 **	79.3 ± 4.0
		19 boys without ASD						
[112]	China 2013	48 children with ASD (mean age of 3.7 ± 1.2 years)	DSM-IV, CARS (all cases)	Season of blood sampling, all study population belonged to Chinese Han population	40:8	0.002	49.8 ± 9.5 **	56.5 ± 11.3
		48 age and sex matched healthy controls (all examined thoroughly by paediatircian for any possible autistic features)						
[118]	Qatar 2014	254 children with ASD (mean age of 5.5± 1.6 years)	ADOS		165:89	0.004	46.0 ± 20.5 **	54.0 ± 21.0
		254 age, sex and ethnicity matched controls						
[79]	Turkey 2014	54 children with ASD (mean age of 59.6 ± 15.0 months)	DSM-IV/ABC-T, ABC^21^ CARS, ADSI, Stanford-Binet or WISC-R	Season of enrolment	47:7	0.069	62.8 ± 28.3 **	52.8 ± 24.3
		54 age, sex, season of enrolment and societal status matched healthy controls						
[108]	Faroe Island 2014	40 individuals with ASD (white European origin of different age) 62 typically developing sibling 77 parents 40 healthy age, sex and season of birth matched controls	ICD-10, DSM-IV/ADOS, DISCO, WISC-III, WAIS-R	Adjustment for month of blood collection	31:9	0.002	24.8 (IQR, 27.5)	37.6 (IQR, 32.3) (controls)
						<0.001		46.1 (IQR, 28.3) (siblings)
						<0.001		46.7 (IQR, 36.2) (parents)
[111]	Egypt 2015	122 children with ASD (mean age of 5.1 ± 1.4 years)	DSM-IV/CARS, ABC ^2^	Blood samples collected during two months	75% male in control group	<0.0001	45.1 ± 21.9 **	106.3 ± 23.7
		100 age, sex and societal status matched healthy controls (all screened for any mental and autistic manifestations)						
[115]	Iran 2015	13 children with ASD (3–12 years)	DSM-IV/CARS		11:2	0.35	13.0 (IQR, 9.6–19.5)	12.0 (IQR, 4.9–13.2)
		14 age and sex matched controls						
[78]	Sweden 2015	58 multi-ethnic children with ASD diagnosed at the age of 4 or older	Multi-professional expert team		51:6	0.01	24.0 ± 19.6	31.9 ± 27.7
		59 healthy siblings						
[117]	China 2016	215 children with ASD (mean age of 4.8 ± 1.0 years)	DSM-IV/ADOS	Blood samples collected during six months	173:42	0.02	-	-
		285 age and sex matched healthy controls (mean age of 5.1 ± 1.1 years)						

* All values reported as means ± standard deviation, otherwise stated; ** The values were ng/mL, they have been converted to nmol/L for easy comparison (1 ng/mL = 2.5 nmol/L); ASD, Autism Spectrum Disorder; 25(OH)D, 25-hydroxyvitamin D; DSM-IV, Diagnostic and Statistical Manual of Mental Disorders-Fourth Edition; US, The United States; ADOS, Autism Diagnostic Observation Schedule; BMI, body mass index; CFD, casein free diet; NCFD, non casein free diet; PDD-BI, Pervasive Developmental Disorder-Behaviour Inventory (autism composite); ATEC, Autism Evaluation Treatment Checklist;; SAS, Severity of Autism Scale; IQR, interquartile range; CARS, Childhood Autism Rating Scale; ADOS, Autism Diagnostic Observation Schedule; ABC-T, Aberrant behaviour checklist-Turkish version; ABC^1^, Autism Behaviour Checklist; ADSI, Ankara Developmental Screening Inventory; WISC-R, Wechsler Intelligence for Children-Revised;.ICD-10, International Classification of Disease-Tenth Edition; DISCO, Diagnostic Interview for Social and Communication Disorders; WISC-III, Wechsler Intelligence Scale for Children-Third Edition; WAIS-R, Wechsler Adult Intelligence Scale-Revised; ABC^2^, Aberrant Behaviour Checklist.
